# One-time fertilization of controlled-release urea with compound fertilizer and rapeseed cake maintains rice grain yield and improves nitrogen use efficiency under reduced nitrogen conditions

**DOI:** 10.3389/fpls.2023.1281309

**Published:** 2023-10-10

**Authors:** Yajie Hu, Qin Cai, Yi Xu, Jiantao Xue, Enwei Yu, Haiyan Wei, Ke Xu, Zhongyang Huo, Hongcheng Zhang

**Affiliations:** Jiangsu Key Laboratory of Crop Cultivation and Physiology, Agricultural College of Yangzhou University/Co-Innovation Center for Modern Production Technology of Grain Crops, Yangzhou University, Yangzhou, China

**Keywords:** rice, reduced nitrogen, one-time fertilization, grain yield, nitrogen use efficiency

## Abstract

Nitrogen (N) rate reduction and simplified fertilization can mitigate environmental impacts and reduce the involvement of manual labor in rice (*Oryza sativa* L.) production. Controlled-release urea (CRU) has been recommended as an effective alternative technique to conventional urea fertilization, and it can improve rice yield and N use efficiency (NUE) and reduce labor costs. However, the information on the effects of one-time fertilization with CRU on maintaining yield and improving NUE under reduced chemical N conditions is limited. In this study, controlled-release bulk blending fertilizer (CRF), consisting of CRU with release periods of 40 and 100 days, mixed with compound fertilizer, was applied as basal fertilizer. Increased ~20% plant density (ID) and rapeseed cake fertilizer (RC, increase 20% organic N) were combined with CRF, respectively. The N treatments with 20% chemical N reduction were as follows: reduced N fertilizer (RNF), CRF, CRF+ID, and CRF+RC. In addition, a conventional split fertilizer application with 300 kg ha^-1^ N was applied as the control (CK). Rice yield and its components, dry matter accumulation, N uptake, and NUE were investigated to evaluate whether one-time N fertilization realized stable yield and high NUE under reduced 20% chemical N conditions. Compared with CK, the CRF+RC treatment exhibited a comparable grain yield, while the other reduced N treatments (RNF, CRF, and CRF+ID) had a lower grain yield. Moreover, CRF+ID exhibited a higher rice grain yield than RNF or CRF under the same N level. Irrespective of exogenous organic N, CRF+RC exhibited significantly higher NUE than CK. The CRF+ID treatment showed a significantly higher N partial factor productivity (PFN) than CK but comparable N agronomic efficiency (NAE) and N recovery efficiency (NRE). Therefore, a one-time fertilizer application of CRF+RC maintained grain yield and improved the NUE while reducing the N rate and fertilization times, demonstrating its potential application in rice production.

## 1 Introduction

Rice (*Oryza sativa* L.) is a basic cereal crop worldwide and the primary staple food for over half of the world’s population (Yuan et al., 2021). Rice yield needs to increase by ~30% to keep up with the rising global population (Godfray et al., 2010; Chen et al., 2014). Therefore, it is vital to ensure rice production for meeting the ever-increasing demands of global food. Expanding the planting area and enhancing the grain yield per unit area are sustainable ways to increase rice production. However, the spread of rice-cultivated land is limited owing to urbanization, soil salinization, water shortages, and climate change (Yu et al., 2012; Liu et al., 2013). Thus, increasing grain yield per unit area is a more effective approach for enhancing rice productivity.

Nitrogen (N) plays the most dominant role in maintaining crop productivity (Grant et al., 2012). To achieve higher grain yields, high N input rate is applied; however, this leads to soil acidification, nitrous oxide emissions, and water pollution (Xia et al., 2017). To reduce the N apparent surplus and improve rice N use efficiency (NUE), N fertilization is generally applied in three to five splits through manual labor to match the N requirements (Zhang et al., 2013; Zhou Y. et al., 2022). However, with the shortage of labor, the multiple applications of N increase the economic cost of artificial fertilization and cannot meet the demands of modern crop production (Cui et al., 2022). Therefore, applying high-efficiency and environmentally friendly N fertilization methods with reduced N fertilizer (RNF) and low topdressing costs is crucial to improve rice yield and grain quality and to minimize N risk to the environment.

In recent years, several improved N fertilizer management techniques have been adopted to enhance rice grain yield, promote NUE, and minimize N fertilizer consumption. These techniques include N rate reduction with dense planting (Chen et al., 2021; Wei et al., 2021), the combined application of organic manure and chemical fertilizer (Zhou et al., 2022), and side-deep fertilization (Li et al., 2021; Min et al., 2021; Chen et al., 2022). Moreover, studies have explored simplified N fertilizer application by relying on slow or controlled-release fertilizer in crops (Guo et al., 2017; Chu et al., 2018; Cheng et al., 2022; Xu et al., 2022). However, a single basal application of slow or controlled-release N fertilizer may not be sufficient to meet the N requirements of rice throughout the entire growth period. To solve this problem, combining the basal application of slow or controlled-release N fertilizer with N topdressing can improve rice yield and NUE (Ke et al., 2017; Lyu et al., 2021a). Several studies on field crops, including rice (Xu et al., 2021; Lyu et al., 2021b; Ding et al., 2022), wheat (Chen et al., 2017; Fan et al., 2021), and maize (Fan et al., 2021; Jiang et al., 2022), have investigated the slow or controlled release of N fertilizer mixed with conventional urea (CU) to improve yield and NUE and reduce N fertilization times. In this study, for N rate reduction and simplified fertilization, we adopted a novel one-time fertilizer application method involving the application of controlled-release urea (CRU) fertilizer to replace tiller and panicle N fertilizer with different release times, combined with the basal application of conventional compound fertilizer. Additionally, we replaced 20% of inorganic N fertilizer with organic fertilizer to achieve reduced inorganic N fertilizer management in rice production. Previous studies had reported that the combined application of organic and chemical N fertilizers improved rice production and promoted N accumulation and use (Zhang et al., 2018; Lin et al., 2023). The fertilizer of rapeseed cake (RC) is one of the important organic fertilizers that can increase grain yield and NUE by improving soil organic matter and the abundance of microorganisms in the soil (Zhou TY. et al., 2022). Meanwhile, we evaluated whether a ~20% increase in planting density combined with CRF could compensate for rice yield loss and enhance NUE under a 20% reduced N rate. Hence, the objective of this study was to investigate the prospect of meeting N demand and obtaining a stable yield and high NUE throughout the entire growth stage of rice using a novel one-time fertilizer application method under reduced N conditions.

## 2 Materials and methods

### 2.1 Experimental site and growth conditions

Field experiments were conducted in Xinghua, Jiangsu, China (33°05′N, 119°58′E), in 2019 and 2020. The cropping system employed a rice-wheat rotation in this study. The physicochemical properties of the 0–20 cm layer of the loamy clay soil were as follows: 26.7 g kg^−1^ organic matter, 1.87 g kg^−1^ total N, 13.40 mg kg^−1^ Olsen-P, and 150.60 mg kg^−1^ available K. The temperature and sunshine hours were measured during the rice-growing seasons of 2019 and 2020 (**Table 1**).

**Table 1:** Meteorological data of rice-growing seasons in 2019 and 2020 in Xinghua, Jiangsu, China.

Month	Average temperature (°C)		Average maximum Temperature (°C)		Average minimum temperature (°C)		Sunshine hours (h)	
	2019	2020	2019	2020	2019	2020	2019	2020
June	24.52	25.13	29.46	29.07	20.49	21.91	156.60	114.30
July	27.72	25.22	31.92	28.58	24.41	22.68	146.10	80.70
August	27.38	29.45	31.26	33.28	24.14	26.57	200.00	174.70
September	23.06	23.48	27.41	28.20	19.31	19.64	165.10	188.50
October	17.52	16.58	22.86	21.45	13.30	12.33	150.60	156.30

### 2.2 Experimental material and design

An inbred *japonica* rice cultivar, Nangeng 9108, which is widely grown on a large-scale production, was used in the experiment. Seedlings were raised in plastic nursery trays. They were sown on May 26, 2019, and May 22, 2020, and transplanted by artificial simulative machine planting on June 20, 2019, and June 16, 2020. Resin-coated urea with 40 and 100 days of controlled release was used in the experiment. The N treatments with 20% N reduction were as follows: reduced N fertilizer (RNF), controlled-release bulk blending fertilizer (CRF), CRF+increased density (ID, increase ~20% planting density), and CRF+RC (increase 20% rapeseed cake fertilizer). The contents of N, P and K are 4.00%, 0.95%, and 1.02% in the rapeseed cake fertilizer in this study. According to local large-scale rice production practices, conventional N amount 300 kg ha^-1^, including five topdressing, and transplanting density 25.65×10^4^ ha^−1^, was set as CK. The more details of management practices in different N treatments are shown in **Table 2**. Phosphorus (90 kg ha^−1^ P) and potassium (90 kg ha^−1^ K) were applied as basal fertilizer in the 0 N treatment. The experiment plot area was 30 m^2^ (5 m × 6 m), with three replications. The plots were separated by a soil ridge with plastic film. Water management and the control of diseases, pests and weeds were implemented according to the requirements of large-scale rice production.

**Table 2:** Details of crop management practices in different nitrogen fertilizer treatments.

Treatment	N rate (kg·ha^−1^)	Density (×10^4^·ha^−1^)		Basal fertilizer (kg·ha^−1^)				Tillering fertilizer (kg·ha^−1^)	Spikelet fertilizer (kg·ha^−1^)	Number of N fertilizer application
				Types of controlled-release urea fertilizer		Compound fertilizer (N)	Rapeseed cake fertilizer (N)	Urea	Urea	
				40 d	100 d					
0N	0	25.65		0	0	0	0	0	0	0
CK	300	25.65		0	0	90	0	60 + 60	60 + 30	5
RNF	240	25.65		0	0	90	0	45 + 45	40 + 20	5
CRF	240	25.65		60	90	90	0	0	0	1
CRF+ID	240	30.32		60	90	90	0	0	0	1
CRF+RC	240 + 60*	25.65		60	90	90	60	0	0	1

0N, no N fertilizer; CK, conventional N fertilization; RNF, reduced N fertilizer; CRF, controlled-release bulk blending fertilizer; ID, increased density; RC, rapeseed cake. *This means that the N rate in the CRF+RC treatment includes chemical and organic N fertilizer.

### 2.3 Sampling and measures

Thirty rice plants in each plot were tagged to count the number of stems and tillers at the middle tillering stage (MT, 20 days after transplanting), jointing stage (JS), heading stage (HS), and maturity stage (MS).

Plants from five hills were sampled from each plot for leaf area and aboveground biomass measurement at JS, HS, and MS. The leaf area was calculated based on leaf length, leaf width, and coefficient (0.75). The leaf area index (LAI) was then calculated. The decreasing rate of leaf area was calculated by the formula. Decreasing rate of leaf area (LAI·d^−1^) = (LAI at HS – LAI at MS)/the days from HS to MS. Plants from five hills were divided into leaves, stems, and panicles (after heading) at JS, HS, and MS. Each sample was separately oven-dried in bags at 105°C for 30 min and then at 80°C to a constant weight. The dried samples were weighed to determine the aboveground biomass.

The dried samples were ground to powder and sieved. The samples were digested with H_2_SO_4_-H_2_O_2_, filtered, and separated into constant-volume samples. The N content was determined by micro Kjeldahl digestion to calculate the aboveground N uptake. The equation was used to calculate the NUE according to Xue et al. (2013). The parameters of NUE are calculated using the following formulas:


$$\begin{array}{c} \text{N Recovery Efficiency}\;(\text{ARE},\%)\\ =\;(\text{Total plant N uptake in N Treatment}\\ -\text{Total plant N uptake in}\;0\;\text{N treatment})/\text{N rate}, \end{array}$$

$$\begin{array}{c} \text{N Agronomic Efficiency}\;(\text{AEN},{\text{kg kg}}^{-1})\\ =(\text{Grain yield in N treatment}-\text{Grain yield in}\;0\;\text{N treatment})/\text{N rate}, \end{array}$$

$$\text{N Partial Factor Productivity}\;\left(\text{PFN},{\text{kg kg}}^{-1}\right)=\text{Grain yield in N treatment}/\text{N rate}.$$

At maturity, a 10 m^2^ area in the middle of each plot was harvested. The rice grains were dried and adjusted to 14.5% moisture to determine the grain yield. Fifty consecutive representative plants in five rows were selected to investigate the panicle number, spikelet per panicle, and seed-setting rate. Additionally, 1000 full grains were weighed to calculate the grain weight.

### 2.4 Statistical analysis

The data were analyzed using Microsoft Excel 2016 and SPSS 17.0 (SPSS, Chicago, IL, USA). The figures were processed using Origin 8.5 (OriginLab, Hampton, MA, USA). The analysis of variance and mean comparison were based on the least significant difference test at the *P*< 0.05 probability level.

## 3 Results

### 3.1 Yield and its components

**Figure 1:**
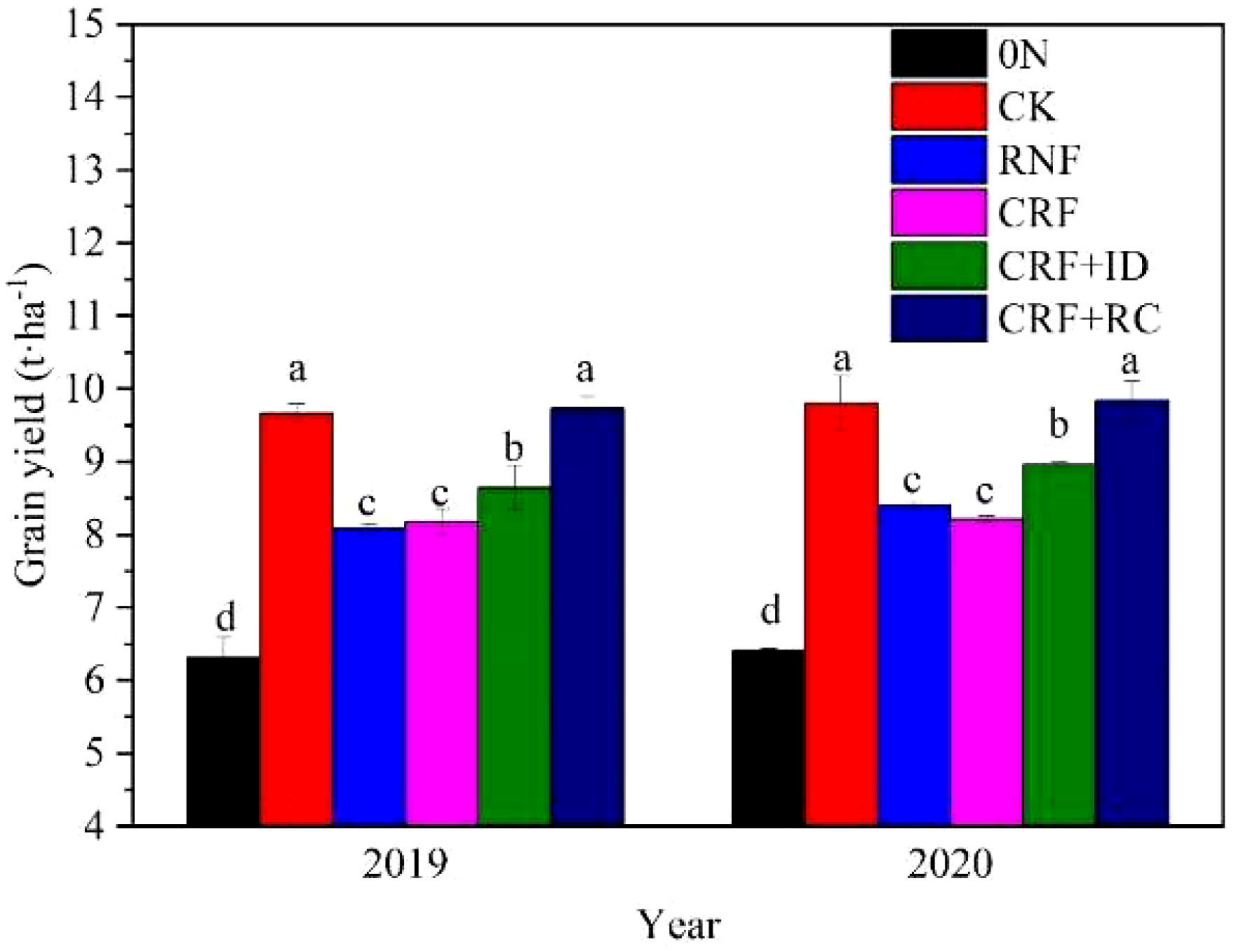
Effects of reduced N and simplified N fertilizer on rice grain yield. 0N, no N fertilizer; CK, conventional N fertilization; RNF, reduced N fertilizer; CRF, controlled-release bulk blending fertilizer; ID, increased density; RC, rapeseed cake. Bars represent the standard deviation (SD). Different letters indicate a significant difference (*P*< 0.05).

The N treatments significantly influenced the grain yield in both years (**Figure 1**). Compared with CK, CRF+RC showed a slight increase in grain yield, while RNF, CRF, and CRF +ID treatments exhibited a significant decrease in grain yield over two years. Under reduced N conditions, CRF showed a yield comparable to RNF, but CRF+ID showed a significantly higher grain yield than RNF. Among the grain yield components (**Table 3**), the panicle number significantly decreased in the reduced N treatments (RNF and CRF) compared to CK, but increased in CRF+RC. Compared with CK, all N treatments exhibited a smaller spikelet number per panicle. In contrast, all N treatments showed higher seed-setting rates than CK. No significant differences in grain weight existed among the N treatments, except for CRF+RC. Hence, the one-time application of CRF+RC treatment achieved a comparable yield to CK, owing to the sufficient number of total spikelets; however, the combination of reducing N by 20% and increasing planting density by ~20% (CRF+ID) did not achieve the same yield as CK.

**Table 3:** Effects of reduced N and simplified N fertilizer on rice yield components.

Year	Treatment	Panicle number (×10^4^ ha^−1^)	Spikelet number per panicle	Total spikelets (×10^6^ ha^−1^)	Seed-setting rate (%)	1000-grain weight (g)
2019	0N	234.60 ± 0.00 d	104.84 ± 0.94 d	245.95 ± 2.21 d	94.65 ± 0.83 a	27.21 ± 0.02 a
	CK	325.13 ± 6.38 ab	129.07 ± 4.65 a	419.92 ± 23.33 a	88.64 ± 0.16 d	26.28 ± 0.10 b
	RNF	297.08 ± 1.27 c	118.35 ± 0.73 bc	351.58 ± 0.66 b	90.56 ± 1.01 c	26.73 ± 0.28 b
	CRF	300.90 ± 2.55 c	114.95 ± 10.74 bc	339.75 ± 29.39 c	92.07 ± 0.30 bc	26.52 ± 0.04 b
	CRF+ID	316.50 ± 4.50 b	111.59 ± 1.41 cd	353.16 ± 9.48 b	93.47 ± 0.20 ab	26.34 ± 0.00 b
	CRF+RC	334.05 ± 0.00 a	124.56 ± 3.87 ab	409.74 ± 12.93 a	91.54 ± 0.30 c	25.39 ± 0.05 c
2020	0N	237.15 ± 0.00 d	106.32 ± 0.42 d	252.15 ± 1.01 d	95.40 ± 0.41 a	27.49 ± 0.47 a
	CK	327.67 ± 6.38 ab	130.84 ± 5.58 a	429.07 ± 26.64 a	89.43 ± 0.13 d	26.43 ± 0.28 b
	RNF	304.73 ± 1.28 c	119.65 ± 0.66 bc	364.63 ± 3.52 b	90.94 ± 0.12 c	26.70 ± 0.17 b
	CRF	304.73 ± 1.28 c	116.42 ± 9.33 bc	348.94 ± 29.92 c	93.01 ± 0.31 b	26.21 ± 0.18 b
	CRF+ID	318.00 ± 6.00 b	113.92 ± 0.17 cd	355.44 ± 7.38 bc	93.10 ± 1.04 b	26.72 ± 0.39 b
	CRF+RC	336.60 ± 2.55 a	126.64 ± 4.36 ab	419.94 ± 17.89 a	91.72 ± 1.18 c	26.57 ± 0.29 b

0N, no N fertilizer; CK, conventional N fertilization; RNF, reduced N fertilizer; CRF, controlled-release bulk blending fertilizer; ID, increased density; RC, rapeseed cake. Values in the same column with different letters are significantly different (P< 0.05). The data represent the mean ± standard deviation (mean ± SD).

### 3.2 Growth period

The difference of rice growth period was found in different N treatments (**Table 4**). Compared to CK, the reduced N treatments (RNF, CRF and CRF+ID) showed a prematurity, but CRF+RC treatment delayed heading and maturity. The whole growth duration was longest in CRF+RC treatment, and extended by 2 days compared with that in CK.

**Table 4:** Effect of reduced and simplified nitrogen fertilizer on growth period of mechanical transplanting rice.

Treatment	SS-TS (d)		TS-JS (d)		JS-HS (d)		HS-MS (d)		Whole growth duration (d)	
	2019	2020	2019	2020	2019	2020	2019	2020	2019	2020
0N	25	25	38	42	28	27	55	53	146	147
CK	25	25	38	42	30	28	58	57	151	152
RNF	25	25	38	42	30	28	57	56	150	151
CRF	25	25	38	42	30	28	57	56	150	151
CRF+ID	25	25	38	42	30	28	57	56	150	151
CRF+RC	25	25	38	42	31	29	59	58	153	154

SS, seeding stage; TS, Transplantation stage; JS, Jointing stage; HS, Heading stage; MS, Maturity stage.

### 3.3 Stem and tiller dynamics

**Figure 2:**
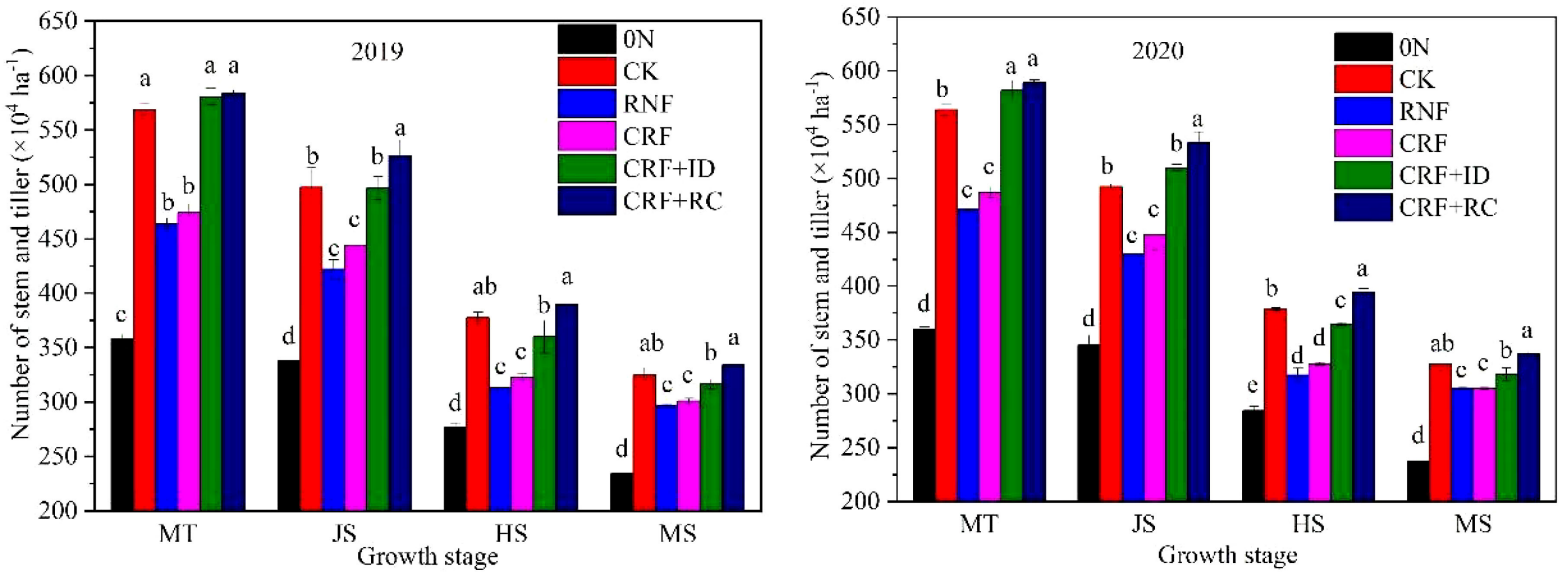
Effects of reduced N and simplified N fertilizer on the stem and tiller dynamics of rice. 0N, no N fertilizer; CK, conventional N fertilization; RNF, reduced N fertilizer; CRF, controlled-release bulk blending fertilizer; ID, increased density; RC, rapeseed cake. MT, middle tillering stage; JS, jointing stage; HS, heading stage; MS, maturity stage. Bars represent the standard deviation (SD). Different letters indicate a significant difference (*P*< 0.05).

The number of stems and tillers at different growth stages was significantly influenced by the N treatments (**Figure 2**). The reduced N treatments (RNF and CRF) considerably reduced the number of stems and tillers at all growth stages compared with CK; however, CRF+ID and CRF+RC significantly increased the number of stems and tillers compared with RNF and CRF. CRF exhibited similar numbers of stems and tillers when compared with RNF. In contrast, the reduced N treatments (RNF and CRF) showed significantly higher productive tiller percentages than CK, CRF+ID, and CRF+RC. These results suggest that reduced N treatment decreased the number of stems and tillers but increased them when supplemented with organic fertilizer or increased planting density. Moreover, at the same N application rate, the one-time application of CRF produced comparable numbers of stems and tillers to RNF at different growth stages.

### 3.4 LAI

The effect of N treatments on LAI was similar to the dynamics of the stem and tiller (**Table 5**). Reduced N treatments (RNF, CRF, and CRF+ID) markedly reduced LAI compared with CK at different growth stages, while the addition of rapeseed cake fertilizer (CRF+RC) resulted in a significantly higher LAI at HS and MS. Moreover, the reduced N treatments increased the decreasing rate of leaf area compared with CK, but there was no significant difference in the decreasing rate of leaf area among the N treatments.

**Table 5:** Effects of reduced N and simplified N fertilizer on the LAI of rice.

Year	Treatment	JS	HS	MS	Decreasing rate of leaf area (LAI·d^−1^)
2019	0N	2.38 ± 0.01 d	4.05 ± 0.09 d	2.18 ± 0.12 d	0.0339 ± 0.00 b
	CK	4.40 ± 0.32 a	7.52 ± 0.05 b	3.62 ± 0.02 b	0.0673 ± 0.00 a
	RNF	3.66 ± 0.16 c	6.85 ± 0.09 c	2.90 ± 0.04 c	0.0693 ± 0.00 a
	CRF	3.86 ± 0.14 bc	6.94 ± 0.12 c	3.06 ± 0.07 c	0.0681 ± 0.00 a
	CRF+ID	3.95 ± 0.14 b	7.47 ± 0.20 b	3.61 ± 0.00 b	0.0678 ± 0.00 a
	CRF+RC	4.31 ± 0.05 a	7.88 ± 0.17 a	3.95 ± 0.10 a	0.0667 ± 0.00 a
2020	0N	2.37 ± 0.10 d	4.16 ± 0.09 d	2.35 ± 0.07 d	0.0341 ± 0.00 b
	CK	4.38 ± 0.07 a	7.52 ± 0.08 b	3.68 ± 0.03 b	0.0673 ± 0.00 a
	RNF	3.75 ± 0.19 c	6.76 ± 0.24 c	3.07 ± 0.23 c	0.0660 ± 0.00 a
	CRF	3.95 ± 0.01 b	6.99 ± 0.03 c	3.26 ± 0.23 c	0.0667 ± 0.00 a
	CRF+ID	3.97 ± 0.06 b	7.60 ± 0.12 b	3.64 ± 0.01 b	0.0707 ± 0.00 a
	CRF+RC	4.38 ± 0.25 a	8.02 ± 0.14 a	3.94 ± 0.16 a	0.0703 ± 0.01 a

0N, no N fertilizer; CK, conventional N fertilization; RNF, reduced N fertilizer; CRF, controlled-release bulk blending fertilizer; ID, increased density; RC, rapeseed cake. JS, jointing stage; HS, heading stage; MS, maturity stage. Values in the same column with different letters are significantly different (P< 0.05). The data represent the mean ± standard deviation (mean ± SD).

### 3.5 Dry matter production and harvest index

The application of N treatments significantly influenced dry matter production (**Table 6**). Compared with CK, the reduced N treatments (RNF and CRF) significantly reduced dry matter weight at JS, HS, and MS; however, the addition of rapeseed cake fertilizer (CRF+RC) led to a comparable dry matter weight at different growth stages. Moreover, the CRF+ID treatment increased the total dry matter weight compared with CRF and RNF, indicating that increasing planting density by ~20% enhanced dry matter production. Similarly, compared with CK, the reduced N treatments reduced dry matter accumulation during the middle and late growth periods, but the CRF+RC treatment showed comparable dry matter accumulation.

**Table 6:** Effects of reduced N and simplified N fertilizer on dry matter weight and period accumulation of rice.

Year	Treatment	JS (t·ha^−1^)	HS (t·ha^−1^)	MS (t·ha^−1^)	JS–HS (t·ha^−1^)	HS–MS (t·ha^−1^)
2019	0N	2.43 ± 0.04 d	7.18 ± 0.03 d	11.69 ± 0.14 d	4.76 ± 0.07 d	4.50 ± 0.11 d
	CK	3.92 ± 0.13 ab	11.07 ± 0.12 ab	17.91 ± 0.79 ab	7.15 ± 0.01 ab	6.84 ± 0.66 ab
	RNF	3.52 ± 0.02 c	10.24 ± 0.21 c	16.74 ± 0.03 c	6.73 ± 0.19 bc	6.49 ± 0.18 c
	CRF	3.63 ± 0.01 c	9.98 ± 0.28 c	16.61 ± 0.28 c	6.34 ± 0.27 c	6.64 ± 0.00 bc
	CRF+ID	3.77 ± 0.02 bc	10.57 ± 0.01 b	17.31 ± 0.38 b	6.80 ± 0.03 bc	6.74 ± 0.38 bc
	CRF+RC	4.14 ± 0.04 a	11.35 ± 0.08 a	18.37 ± 0.09 a	7.20 ± 0.13 a	7.01 ± 0.01 a
2020	0N	2.53 ± 0.07 d	7.25 ± 0.12 c	11.72 ± 0.11 d	4.72 ± 0.05 c	4.47 ± 0.23 c
	CK	3.99 ± 0.09 ab	11.31 ± 0.12 a	18.14 ± 0.32 ab	7.31 ± 0.02 a	6.84 ± 0.43 a
	RNF	3.54 ± 0.04 c	10.49 ± 0.21 b	16.56 ± 0.29 c	6.95 ± 0.17 b	6.07 ± 0.08 b
	CRF	3.66 ± 0.01 c	10.66 ± 0.09 b	16.75 ± 0.30 c	6.99 ± 0.08 b	6.09 ± 0.21 b
	CRF+ID	3.79 ± 0.06 bc	10.56 ± 0.27 b	17.70 ± 0.57 b	6.77 ± 0.21 b	7.14 ± 0.30 a
	CRF+RC	4.16 ± 0.04 a	11.54 ± 0.39 a	18.56 ± 1.01 a	7.37 ± 0.43 a	7.03 ± 0.62 a

0N, no N fertilizer; CK, conventional N fertilization; RNF, reduced N fertilizer; CRF, controlled-release bulk blending fertilizer; ID, increased density; RC, rapeseed cake. JS, jointing stage; HS, heading stage; MS, maturity stage.

Values in the same column with different letters are significantly different (P< 0.05). The data represent the mean ± standard deviation (mean ± SD).

**Figure 3:**
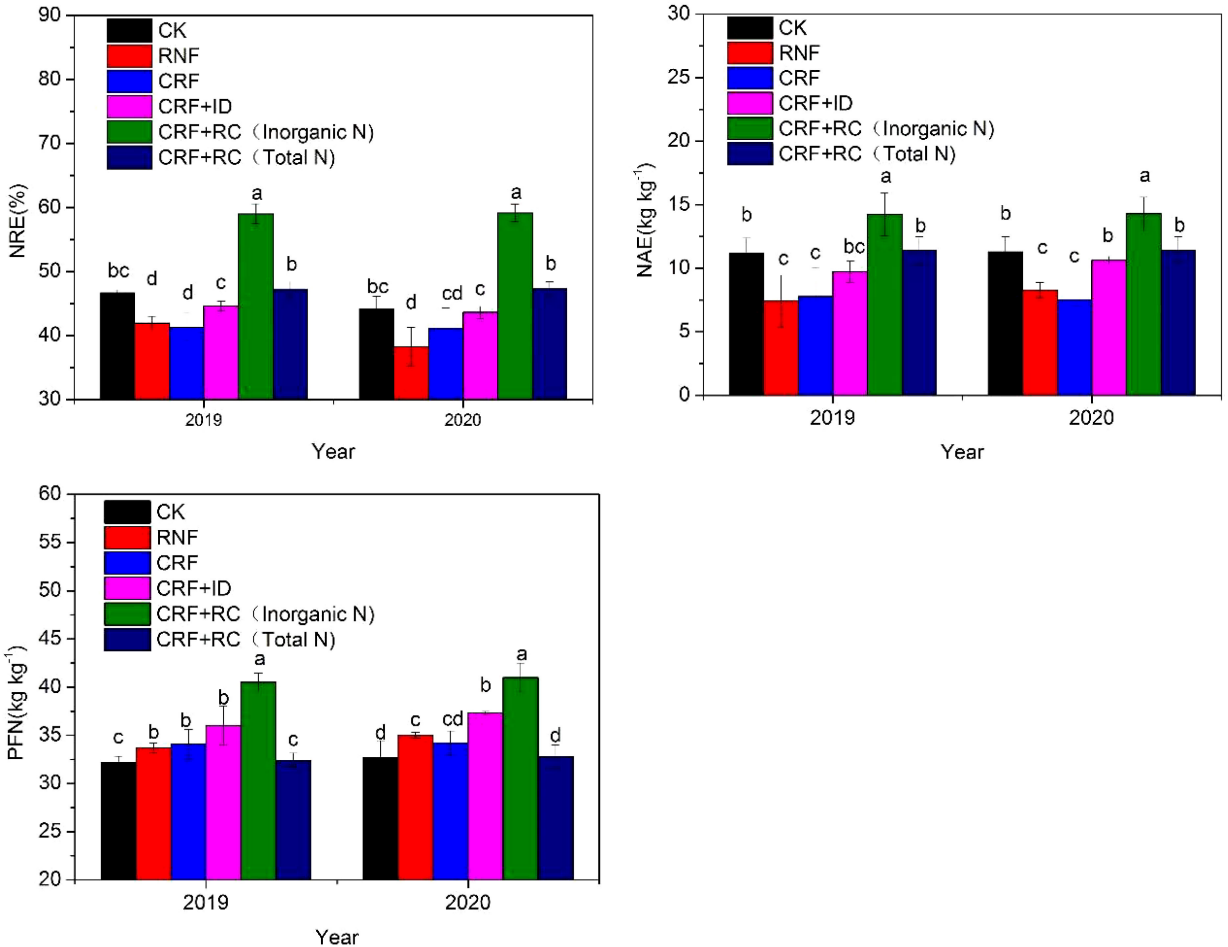
Effects of reduced N and simplified N fertilizer on the harvest index of rice. CK, conventional N fertilization; RNF, reduced N fertilizer; CRF, controlled-release bulk blending fertilizer; ID, increased density; RC, rapeseed cake. Bars represent the standard deviation (SD). Different letters indicate a significant difference (*P*< 0.05).

**Figure 3** shows the difference of harvest index in the different N treatments. Compare with CK, the reduced N treatments (RNF and CRF) significantly reduced harvest index expect for CRF in 2019. Harvest index was higher in CRF+ID and CRF+RC treatments than that in RNF and CRF treatments. And it was comparable between CRF+RC treatment and CK.

### 3.6 N uptake and accumulation

The effect of N treatments on N uptake and accumulation was basically the same as that of dry matter production (**Table 7**). Compared with CK, the reduced N treatments showed significantly lower N uptake at JS, HS, and MS, but there was no difference in N uptake between CRF+RC and CK. Under the same N level, the CRF+ID treatment showed a slightly higher N uptake than the CRF and RNF treatments at different growth stages. For N accumulation, the reduced N treatments reduced the N accumulation from JS to HS compared with CK. The CRF+RC treatment showed higher N accumulation than CK during the middle and late growth periods.

**Table 7:** Effects of reduced N and simplified N fertilizer on nitrogen uptake and accumulation of mechanical transplanting rice.

Year	Treatment	JS (kg·ha^−1^)	HS (kg·ha^−1^)	MS (kg·ha^−1^)	JS–HS (kg·ha^−1^)	HS–MS (kg·ha^−1^)
2019	0N	40.28 ± 0.03 d	64.19 ± 0.33 d	84.03 ± 0.23 d	23.92 ± 0.36 c	19.84 ± 0.09 c
	CK	115.54 ± 2.45 a	192.65 ± 1.08 a	224.00 ± 1.14 a	77.10 ± 1.36 a	31.35 ± 0.06 b
	RNF	87.08 ± 1.07 c	146.71 ± 0.22 c	184.68 ± 1.55 c	59.63 ± 1.28 b	37.97 ± 1.33 ab
	CRF	93.56 ± 2.41 bc	152.71 ± 2.16 bc	183.13 ± 7.34 c	59.16 ± 0.26 b	30.42 ± 9.49 b
	CRF+ID	96.03 ± 2.12 b	155.54 ± 6.29 b	191.08 ± 1.53 b	59.51 ± 8.42 b	35.54 ± 7.82 b
	CRF+RC	113.17 ± 2.05 a	186.71 ± 2.31 a	225.71 ± 2.86 a	73.54 ± 4.36 a	39.00 ± 5.17 a
2020	0N	41.72 ± 2.33 d	67.25 ± 1.50 c	87.57 ± 1.73 d	25.53 ± 0.83 c	20.31 ± 0.23 c
	CK	116.07 ± 1.38 a	194.35 ± 0.44 a	220.07 ± 5.88 a	78.27 ± 0.94 a	25.73 ± 6.32 bc
	RNF	88.23 ± 1.92 c	153.96 ± 5.53 b	179.36 ± 8.09 c	65.73 ± 3.61 b	25.41 ± 13.62 bc
	CRF	94.12 ± 0.58 bc	155.18 ± 9.29 b	186.35 ± 7.02 bc	61.06 ± 9.87 b	31.17 ± 2.27 b
	CRF+ID	96.88 ± 4.94 b	155.93 ± 4.00 b	192.22 ± 3.31 b	59.05 ± 8.94 b	36.29 ± 7.31 a
	CRF+RC	116.83 ± 0.60 a	192.18 ± 0.40 a	229.55 ± 0.55 a	75.34 ± 0.19 a	37.38 ± 0.96 a

0N, no N fertilizer; CK, conventional N fertilization; RNF, reduced N fertilizer; CRF, controlled-release bulk blending fertilizer; ID, increased density; RC, rapeseed cake. JS, jointing stage; HS, heading stage; MS, maturity stage.

Values in the same column with different letters are significantly different (P< 0.05). The data represent the mean ± standard deviation (mean ± SD).

### 3.7 NUE

**Figure 4:**
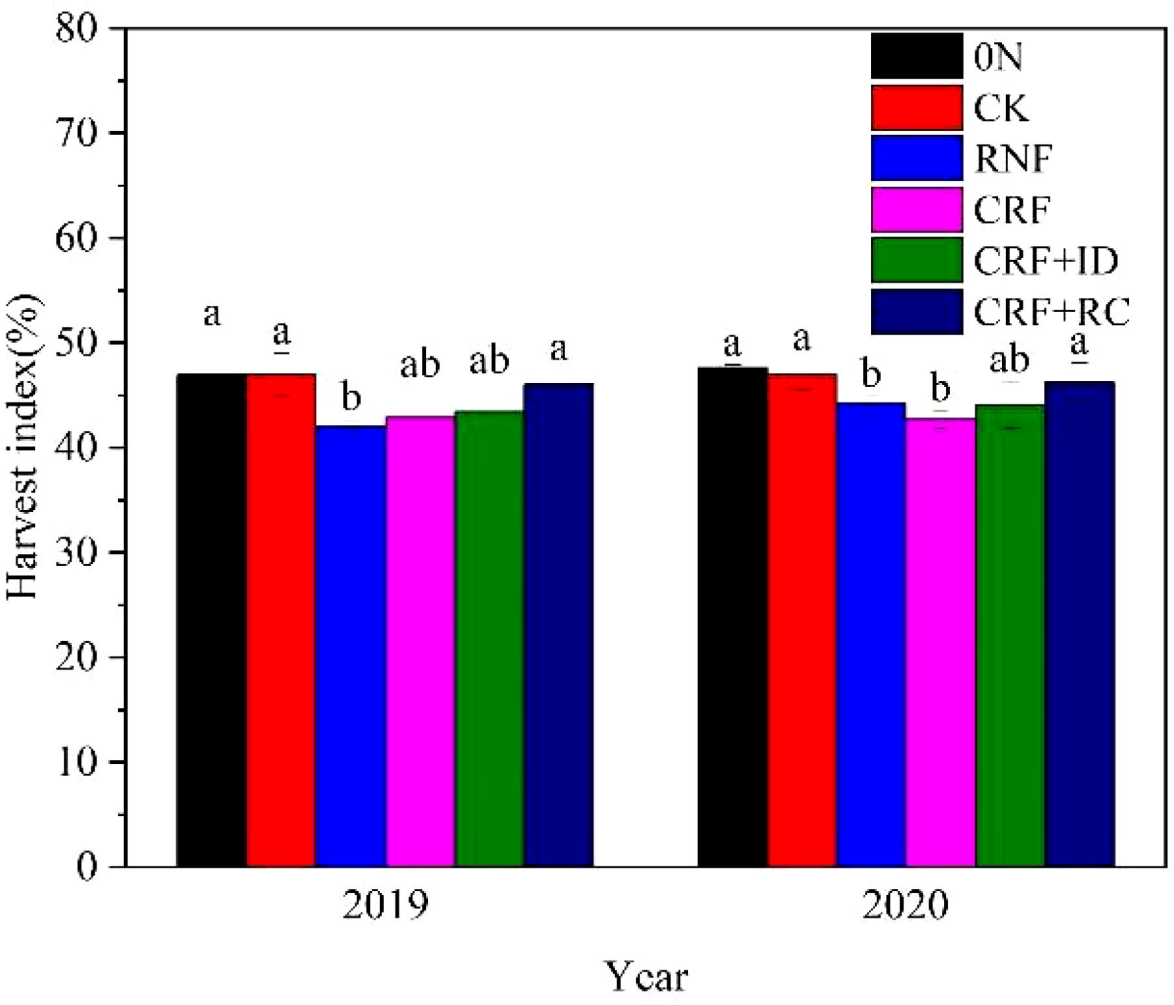
Effects of reduced N and simplified N fertilizer on the N use efficiency of rice. NRE, N recovery efficiency; NAE, N agronomic efficiency; PFN, N partial factor productivity; CK, conventional N fertilization; RNF, reduced N fertilizer; CRF, controlled-release bulk blending fertilizer; ID, increased density; RC, rapeseed cake. Bars represent the standard deviation (SD). Different letters indicate a significant difference (*P*< 0.05).

The N treatments significantly influenced the NUE (**Figure 4**). For inorganic NUE, the CRF+RC treatment showed significantly higher NRE, NAE, and PFN than the other N treatments. For total N (including organic and inorganic N fertilizer input), compared with CK, the CRF+RC treatment showed comparable NUE (NRE, NAE, PFN), but the reduced N treatments (RNF and CRF) showed a significantly lower NRE and NAE and significantly higher PFN. Under the same N level, the CRF+ID treatment showed a higher NUE than the CRF and RNF treatments, indicating that increasing planting density combined with CRF improved NUE under reduced N conditions.

## 4 Discussion

### 4.1 Effects of reduced and simplified N fertilizer application on grain yield and dry matter accumulation

Reducing the N rate and simplifying N management present significant challenges for rice increasing or maintaining grain yield in rice–wheat intensive cropping systems in China. Traditionally, high N rates have been applied to achieve high rice yields in south China, particularly in the middle and lower Yangtze River regions. Frequent N fertilization meets the N absorption requirements of rice but increases labor costs. Hence, one-time fertilization applications have been extensively studied in rice production (Xu et al., 2021; Ding et al., 2022; Zhao et al., 2023). Under the same or reduced N rates, the one-time N fertilization of CRU mixed with normal urea increases the grain yield of rice (Zhao et al., 2023) and wheat (Lu et al., 2022) compared with conventional farmer fertilization practices. In the present study, under the same N rate, the CRF and RNF treatments exhibited comparable rice grain yields, but the one-time application of CRF reduced the number of fertilizer applications by a factor of four compared with the RNF treatment, indicating that the one-time fertilization application could achieve a comparable grain yield under the same N level. Reduced N rates generally result in lower grain yield in crops. However, increasing planting density, rapeseed cake fertilizer, or organic fertilizer is a potential method for reducing the N application rate while offsetting rice grain yield loss. Wei et al. (2021) reported that reducing the N application rate by 15% while increasing planting density by 20% achieved a similar grain yield for japonica inbred rice compared with the control but significantly reduced grain yield for indica hybrid rice. In this study, compared with CK, reducing the N rate by 20% while increasing planting density by ~20% (CRF+ID treatment) significantly reduced grain yield, while the CRF+RC treatment (using rapeseed cake fertilizer to replace 20% inorganic N rate) achieved comparable grain yield. Shi et al. (2021) also reported that replacement of 20% chemical N fertilizer with organic fertilizer allowed for conserving soil fertility and achieving high yield. Hence, increasing planting density by ~20% could not offset the loss of rice yield under a 20% reduction in chemical N fertilizer, but the use of organic fertilizer to replace 20% of chemical N fertilizer resulted in a comparable grain yield compared with CK.

Rice yield is closely associated with dry matter production, particularly during the middle and late growth periods. In this study, dry matter weight and accumulation were significantly lower under reduced N treatments (RNF, CRF, and CRF+ID treatments) than under CK but slightly higher under the CRF+RC treatment. Hence, the effect of organic fertilizer replacement on dry matter production was superior to that of ID under RNF application. Studies have attributed the high dry matter accumulation to the higher number of stems and tillers, higher LAI at different growth stages, and stronger photosynthetic activity during the middle and late growth periods of rice (Zhao et al., 2021). Compared with CK, CRF+ID showed a rather high number of stems and tillers during MT and JS; however, the CRF+ID treatment showed a lower number of stems and tillers than CK. Moreover, CRF+RC showed a higher number of stems and tillers and a higher LAI than CK. These results suggest that the CRF+RC treatment showed a high number of stems and tillers and LAI, contributing to high dry matter accumulation.

### 4.2 Effects of reduced N and simplified N fertilizer application on N accumulation and NUE

Studies have reported that the application of CRU and CRU mixed with CU reduces N loss and increases N accumulation and NUE in crops (Cui et al., 2022; Zhao et al., 2023). Under the same N application rate, compared with conventional split fertilization, the one-time basal application of 60% CRU + 40% CU improved the NUE by 9.7–12.1% in wheat (Cui et al., 2022). Zhao et al. (2023) found that the one-time basal application of CRU mixed with CU increased N uptake and NUE in rice compared with farmer fertilization practices. In contrast, our results showed that the RNF and CRF treatments exhibited no significant differences in NUE under the same N level, which may be attributable to the different release times of CRU or the CRU–CU mixing ratio. In previous studies, under reduced N conditions, increasing plant density or organic fertilizer increased N uptake and NUE (Murray and Linquist, 2020; Luo et al., 2022). In this study, the CRF+ID treatment improved N uptake and NUE (NRE, NAE, and FPN) compared with RNF and CRF treatments. However, CRF+ID exhibited significantly higher FPN than CK but comparable NRE and NAE. Compared with CK, reduced N treatments (RNF and CRF) significantly reduced N uptake and NUE (NRE and NAE), except for PFN. For inorganic NUE, the CRF+RC treatment exhibited higher NRE, NAE, and PFN than the other N treatments (including CK). Therefore, the one-time basal fertilizer application of CRF+RC treatment could reduce the chemical N rate and improve the inorganic NUE.

## 5 Conclusion

The CRF+RC treatment (CRU mixed with compound fertilizer and rapeseed cake) exhibited a grain yield comparable to CK, while the RNF, CRF, and CRF+ID treatments showed a lower grain yield than CK. The CRF+RC treatment exhibited a higher inorganic NUE than CK. The other reduced N treatments (RNF and CRF) showed significantly lower NUE than CK, except FPN. However, CRF+ID and CK exhibited no significant differences in NUE, except FPN. Hence, the one-time fertilization of CRF+RC treatment could maintain grain yield and improve NUE while reducing the chemical N fertilizer application rate and fertilization times.

## Data availability statement

The original contributions presented in the study are included in the article/supplementary material. Further inquiries can be directed to the corresponding author.

## Author contributions

YH: Writing – original draft, Writing – review & editing, Funding acquisition, Project administration, Resources, Supervision. QC: Data curation, Formal Analysis, Investigation, Software, Writing – review & editing. YX: Data curation, Formal Analysis, Investigation, Software, Writing – review & editing. JX: Data curation, Investigation, Methodology, Project administration, Writing – original draft. EY: Data curation, Investigation, Software, Writing – review & editing. HW: Resources, Software, Writing – review & editing. KX: Resources, Writing – review & editing. ZH: Funding acquisition, Resources, Supervision, Writing – review & editing. HZ: Funding acquisition, Resources, Supervision, Writing – review & editing.
